# Data Sharing in the Post-Genomic World: The Experience of the International Cancer Genome Consortium (ICGC) Data Access Compliance Office (DACO)

**DOI:** 10.1371/journal.pcbi.1002549

**Published:** 2012-07-12

**Authors:** Yann Joly, Edward S. Dove, Bartha M. Knoppers, Martin Bobrow, Don Chalmers

**Affiliations:** 1 International Data Access Committee, International Cancer Genome Consortium, Toronto, Ontario, Canada; 2 Data Access Compliance Office, International Cancer Genome Consortium, Montreal, Quebec, Canada; 3 Centre of Genomics and Policy, Department of Human Genetics, Faculty of Medicine, McGill University, Montreal, Quebec, Canada; 4 Department of Medical Genetics, University of Cambridge, Cambridge, United Kingdom; 5 Faculty of Law, University of Tasmania, Tasmania, Australia; University of California San Diego, United States of America

## Introduction

The scientific community, research funders, and governments have repeatedly recognized the importance of open access to genomic data for scientific research and medical progress [1]–[4]. Open access is becoming a well-established practice for large-scale, publicly funded, data-intensive community science projects, particularly in the field of genomics. Given this consensus, restrictions to open access should be regarded as exceptional and treated with caution. Yet, several developments [5] have led scientists and policymakers to investigate and implement open access restrictions [5]–[9]. Notably, there are privacy concerns within the genomics community and critiques from some researchers that open access, if left completely unregulated, could raise significant scientific, ethical, and legal issues (e.g., quality of the data, appropriate credit to data generators, relevance of the system for small and medium projects, etc.) [1]–[10]. A recent paper by Greenbaum and colleagues in this journal [11] identified protecting the privacy of study participants as the main challenge to open genomic data sharing.

One possible way to reconcile open data sharing with privacy concerns is to use a tiered access system to separate access into “open” and “controlled.” Open access remains the norm for data that cannot be linked with other data to generate a dataset that would uniquely identify an individual. A controlled access mechanism, on the other hand, regulates access to certain, more sensitive data (e.g., detailed phenotype and outcome data, genome sequences files, raw genotype calls) by requiring third parties to apply to a body (e.g., custodian, original data collectors, independent body, or data access committee) and complete an access application that contains privacy safeguards. This mechanism, while primarily designed to protect study participants, can also be used to protect investigators, database hosting institutions, and funders from perceptions or acts of favoritism or impropriety. The experience of controlled access bodies to date has been only minimally documented in the literature [9], [12]. To address this lacuna, we present the experience of the Data Access Compliance Office (DACO) of the International Cancer Genome Consortium (ICGC). The goal is to provide information on this increasingly important type of database governance body.

## ICGC and the Development of Controlled Access Policies

Controlled access mechanisms may be viewed as the product of dual imperatives: 1) the legal and ethical requirements of regulators and research ethics committees, as well as research funders and study participants, to protect the confidentiality of data from re-identification and misuse by third parties; and 2) pressure, largely from within the science community, to protect data-producing investigators from acts of free riding by other members of the community (e.g., by ensuring they are properly acknowledged in publications and that no parasitic patents are deposited on the data by subsequent data users). Both issues have been described elsewhere in greater detail [13], but we note that known cases of abuse to date have been rare and so far resolved swiftly by the scientific community [14], [15]. Nevertheless, the publication of the Lin et al. paper, which demonstrated that an individual could be uniquely identified with access to just 75 single-nucleotide polymorphisms from that person [16], and the Homer et al. paper, which demonstrated that knowing even some genetic information about an individual could lead to that individual being identified as belonging to the control or affected group within a study [17], prompted some researchers to suggest that “it [would] be more appropriate to release genome data into databases with restricted access” [18].

Early models of databases having a two-tiered open/controlled access system included the database of Genotypes and Phenotypes (dbGaP) at the US National Institutes of Health (http://www.ncbi.nlm.nih.gov/gap), the Wellcome Trust Case Control Consortium (WTCCC) (http://www.wtccc.org.uk/), the Malaria Genomic Epidemiology Network (MalariaGEN) (http://www.malariagen.net/), and the European Genome-phenome Archive (EGA) (https://www.ebi.ac.uk/ega/). ICGC, a large-scale initiative launched in 2008 to analyze 25,000 cancer genomes, followed this two-tiered system because the data produced by ICGC member projects included prospective and retrospective cohorts of cancer patients, substantial clinical annotation, and coded genomic data. ICGC also adopted at the outset policies for extensive data sharing with the scientific community. The consortium is still growing, but already, 47 project teams in 15 international jurisdictions have initiated studies of over 18,000 tumor genomes.

**Figure 1:**
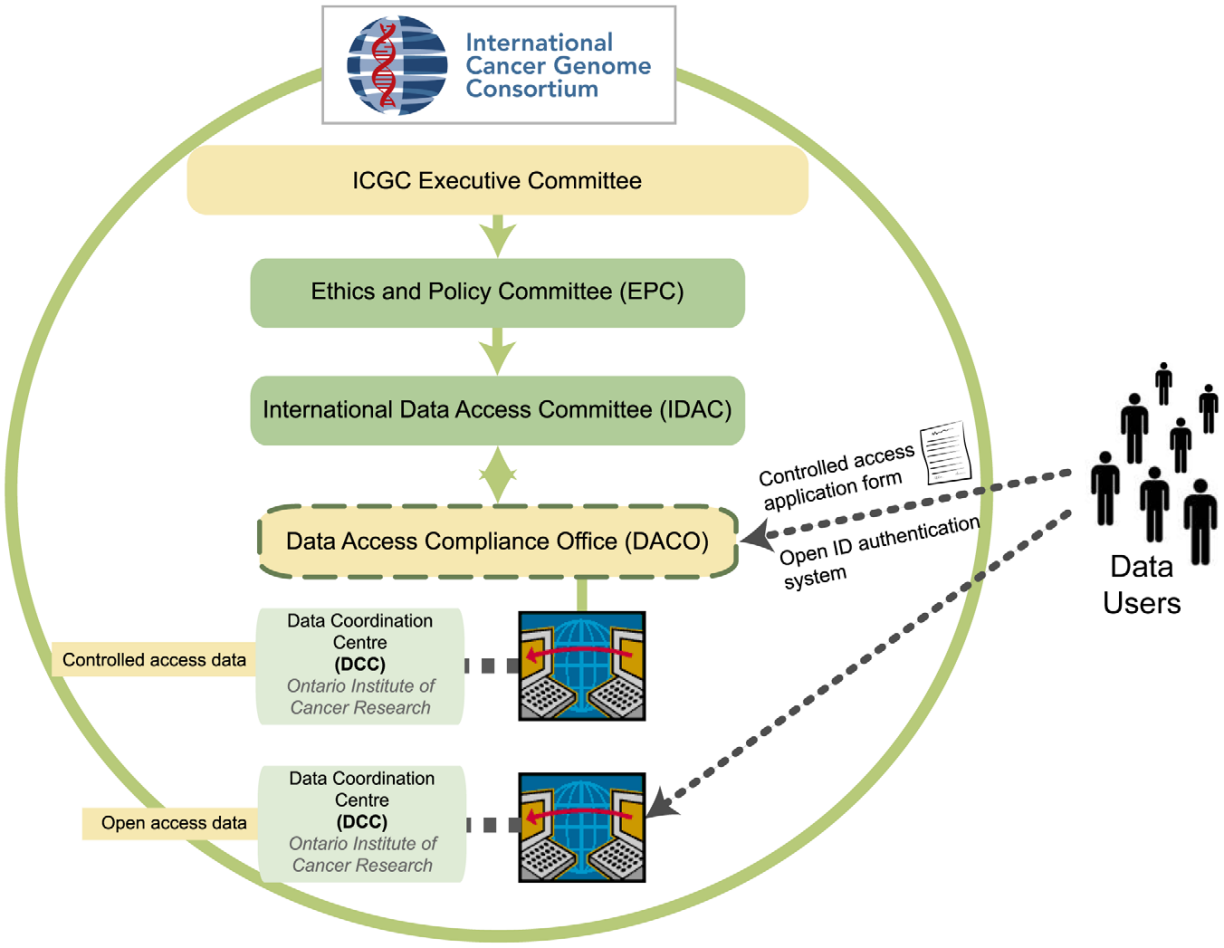
ICGC-DACO tiered access process and storage of controlled ICGC data. The Ontario Institute for Cancer Research (OICR) in Toronto is the host of the ICGC Data Coordination Center (DCC). All ICGC controlled access data is hosted at DCC with the exception of the raw sequence reads (BAM files), which are currently hosted at the European Bioinformatics Institute's (EBI) European Genome-phenome Archive (EGA). Access to these files is coordinated with DCC once DACO approval is granted.

In developing its foundational policy, Goals, Structures, Policies and Guidelines, ICGC drafted and publicly disseminated a comprehensive list of data that would be deposited in each of the open and controlled access categories [19]. This ICGC list was not considered permanently fixed, so that certain types of data could be transferred from the open to the controlled category and vice versa in response to developments in the scientific, technical, legal, and ethical issues previously discussed. For example, following publication of the Homer et al. paper [17], ICGC moved its aggregate genome data from open to controlled access. Importantly, ICGC established two bodies to oversee controlled access: DACO and an International Data Access Committee (IDAC). DACO is responsible for processing access requests from the scientific community and its activities are overseen by IDAC. DACO is required to verify the conformity of users' projects with the goals and policies of ICGC, including, but not limited to, policies concerning the purpose and relevance of the research, the protection of participants, and the security of participants' data. DACO, IDAC, and ICGC's Ethics and Policy Committee (EPC) (Figure 1) collaboratively developed the data access application forms (which include an access agreement), as well as the policies to be used by ICGC. The rules and policies of ICGC have influenced the controlled access strategies of several database projects, including the Wellcome Trust Sanger Institute (http://www.sanger.ac.uk/) and the Human Epigenome Consortium (http://www.epigenome.org/).

## Controlled Access Forms: A Constant Work in Progress

DACO's first version of a data access application was based on a commercial material transfer agreement (MTA). However, the drafting group perceived this version as overly legalistic and impractical. It was never used and was replaced with a simpler access form adapted from the few databases' controlled access policies that were publicly available at the time. The DACO access form (available upon simple login at http://www.icgc.org/daco) comprises two main sections. The first section requires the applicant to provide basic personal (e.g., mailing and e-mail address) and project (e.g., institution, scientific abstract) information. The second section, the “access agreement”, consists of the conditions users must abide by to access ICGC controlled data (e.g., no parasitic patents on primary data, no attempts to re-identify study participants, etc.). Since it was first developed, DACO has circulated the access form to collaborators in various international scientific institutions for feedback. Both the principal investigator requesting controlled access data and an institutional representative are required to sign the access form. DACO considered that this administrative requirement would improve the quality of the data provided and bind both the data user and its institution to the conditions of the access agreement [8].

Since the launch of the controlled access tier, DACO has periodically reviewed the access form to address specific issues and rationalize the process for members of the scientific community. For example, the information technology (IT) security section no longer requires applicants to make an extensive assessment of their IT security policies. Instead, it advises data users of good IT security practices and requires them to certify that they will be using such practices to protect ICGC controlled data. However, as a tradeoff for this user-friendly approach, DACO reserves the right to audit users' IT security documents, if warranted by the circumstances. Although challenging from an IT management standpoint, it may be worth considering the adoption of multiple levels of security and access in the future (i.e., greater control when data re-identification is simple, greater freedom for other types of data where considerable effort and sophistication is required for re-identification). This could constitute an even more flexible approach that would account for different types of controlled data. DACO will continue to regularly revise and update the access form. Indeed, other sections of the form are currently undergoing review, including the requirement for users to provide a valid Open ID (used as an identifier to access the controlled data) and the ethical compliance section.

## The Ethics Section of the Access Form

The ethics compliance section is a key section of the access form for several reasons. First, ethical standards for the use of coded genomic data from international databases vary considerably from country to country [20]. Second, according to ICGC policy, member projects must indicate in their consent documents that the study participants' coded information will be shared with the international research community and that ethics approval for this sharing was obtained. Third, IDAC's opinion has always been that DACO itself is not constituted as an ethics review committee and should not evaluate users' consent forms or research protocols. Essentially, IDAC and DACO rely on the local ethics processes of the data users without imposing another layer of ethics review requirements on them.

However, ICGC members are committed to the need for data users to be cognizant of, and comply with, the ethical policies applicable in their country. Accordingly, if required locally, ethical approval to access and use ICGC controlled data must be obtained. Only in this situation, DACO ensures that this requirement is met by asking for a copy of the ethics approval letter. In cases where a data user is in doubt as to the ethical standards applicable to his research, DACO will encourage him to contact a local ethics committee for guidance. DACO statistics (Table 1) show that a large majority of users indicate that their project does not require formal ethics approval. Initially, data users could also decide to supply an ethics waiver, usually in the form of an official letter from their local ethics committee indicating that their project did not need to be reviewed. This option created confusion and DACO has decided to remove it to facilitate the application process by limiting the available options to two statements: 1) “my country/region does not require my project to undergo ethics review”; or 2) “my country/region requires my project to undergo ethics review”. This simplification was included in a revised version of the ethics section of the DACO Application for Access to ICGC Controlled Data form implemented at the ICGC 6th Scientific Workshop in March 2012.

**Table 1:** Statistics on DACO applications for controlled access to ICGC data (current as of June 15, 2012).

Information	Statistics
Operational launch of the DACO site	July 2010
Number of DACO-approved projects	19
Number of DACO-approved users	123
Number of rejected projects or users	0
Average time to process a new application (based on the last 5 applications processed)	7 calendar days
Average time to process a re-submitted application (based on the last 5 applications processed)	3 calendar days
Number of DACO-approved projects that did not require local ethics committee approval	18
Number of DACO-approved projects that required local ethics committee approval	1
Average time from approval of DACO application to gain access to controlled data through Data Coordination Centre (DCC) portal and European Genome-phenome Archive (EGA)	<24 hours
Average time to gain access to ICGC raw sequence reads (BAM files) held at EGA, from download request to availability of files	3–5 business days

The ethics section of the access form, like the IT security section, is predominantly based on principles of self-reporting and good faith. While perhaps open to critique by legal purists, ICGC and DACO think that this approach relies on the capacity of the scientific community to police itself without raising the administrative burden to an unacceptable level for both data producers and users. The access form remains a legally binding document, but by keeping legal language and requirements to a minimum, DACO aims to minimize the potentially strong negative impact of legal recourses on users (not to mention the difficulty of obtaining such recourses in an international forum), and instead rely on community sanction mechanisms [8]. The ethics section is innovative as few international genomics database projects specifically incorporate an ethics section in their controlled access documents. Other projects usually address ethics concerns through specific conditions in the access agreement (e.g., the prohibition on re-identifying study participants and transferring the data to unauthorized users, etc.) and generally avoid use of the term “ethics” itself. ICGC members consider that a specific ethics section concretely raises the level of protection to meaningfully respond to privacy issues and can foster participants' trust in genomic research. Nevertheless, future empirically driven studies may demonstrate that privacy could be better addressed through a different type of governance arrangement. Recent findings also indicate that perfect anonymity in genomic research, like the risk of significant data misuse, is limited [21], [22]. IDAC strives to remain informed of users' satisfaction with the controlled access process; to this end it will soon undertake a short users' survey and include a few optional questions on the DACO Annual Renewal Application form.

## DACO@Work

Since the first controlled datasets were deposited in the ICGC controlled access database in January 2011, encouraging the various member projects to release their controlled access data in a timely manner has been a challenge and a priority. Consequently, the amount of datasets in the controlled access database is expected to increase significantly in the coming months. To illustrate the difference between controlled and open access, Greenbaum and colleagues presented a comparison between controlled access at ICGC and open access at the Personal Genome Project (PGP) [11].

While legal scholars Anna Pigeon and Anne Cambon-Thomsen have included an interesting critique of the Greenbaum et al. paper in a currently unpublished GEN2PHEN project (http://www.gen2phen.org) report for the European Union (EU) Commission, we wish to highlight here the very different nature of ICGC and the PGP that were compared in the paper. ICGC is an international project of researcher-generated cancer patient databases (including data from both retrospective and prospective patient cohorts), while the PGP is a public scientists' collaborative project with direct self-deposit by individual volunteers. Given these significant differences, the comparison may be uninformative. In addition, Greenbaum and colleagues' methodology for comparison is not explained and it may create confusion by comparing the number of *viewers* on the PGP website to the number of *projects authorized for access* to ICGC controlled data. As the ICGC model is new and still developing, it may be more helpful to look to a similar project such as The Cancer Genome Atlas (TCGA, http://cancergenome.nih.gov/), whose statistics indicate that about 13% of all data requested is controlled data, with the majority of those controlled data requests seeking data on glioblastoma multiforme (*n* = 794) and serous cystadenocarcinoma (*n* = 513).

To date, DACO has approved 123 users from 19 research projects for access to controlled data and has not rejected any access requests submitted to its attention (Table 1). However, some users were asked to re-submit their application form before they could be authorized due to inaccurate or ambiguous information. Applications have so far been processed within a week of electronic submission, but approval time may lengthen as the application rate increases. While the number of approved controlled access requests appears small, it is partially attributable to the limited number of controlled datasets available. As more data becomes available, DACO expects the number of access requests to increase. Because of differing circumstances and variables, it is difficult to accurately compare ICGC open and controlled access levels of activity. However, statistics concerning the open data portal (welcoming over 8,300 unique visitors since June 5, 2011) suggest that the number of users of open access ICGC data is substantially higher. This is partly attributable to the fact that many more open access datasets are currently available through the ICGC data access portal than controlled access datasets. The number of visitors on the open access portal also likely includes a number of internal users (e.g., ICGC quality control and troubleshooting specialists) and curious scientists and journalists who may not actually want to use the data.

## Concluding Remarks

There is certainly an economic and social cost to implementing a controlled access strategy in a genomic database consortium. From the consortium's perspective, financial and intellectual capital must be invested in IT, human resources, and regulatory procedures. From the data user's perspective, completing a multi-page access form can be perceived as a burden. Researchers are already subjected to numerous administrative requests from research funders, academic institutions, and research ethics committees [23]–[26]. Obtaining a signature from an institutional representative and determining one's ethical obligations in a complex research endeavor can be time consuming, although we suggest the time invested is likely to be only a very small part of the effort devoted to any serious research project.

Yet, a well-crafted controlled access strategy could also encourage data producers, especially those working in biomedical research fields where open sharing is still a recent phenomenon, to deposit more data for sharing at a faster pace. It is also possible that as the scientific community familiarizes itself with the controlled access process, data access applications and agreements will become more streamlined and user friendly. More importantly, controlled access offers the possibility to alleviate documented privacy concerns [27], [28] while offering an extra layer of protection to study participants, scientists, and academic institutions. This protection may not be supported by empirical data on the frequency or importance of privacy breaches, but is still warranted during this period of technological transition (e.g., digitization of health records and their sharing across the research and health professions, growth of open sharing in biological sciences, etc.), which poses an unprecedented challenge to the confidentiality of health information (including biological and phenotype data).

Ultimately, the medical privacy debate reflects concerns that go beyond the specific context of genomic research. Society may have to re-conceptualize and contextualize medical confidentiality and personal privacy so that they remain relevant in the context of 21st century science and medical practice. As the “new privacy” is taking shape, controlled access mechanisms could have a major role to play as a necessary complement in the context of open genomic databases and other data-intensive scientific fields [13].
